# Fiducial marker position affects target volume in stereotactic lung irradiation

**DOI:** 10.1002/acm2.13596

**Published:** 2022-04-04

**Authors:** Hiroaki Akasaka, Kazufusa Mizonobe, Yuya Oki, Kazuyuki Uehara, Masao Nakayama, Shuhei Tamura, Yoshiki Munetomo, Haruna Kawaguchi, Jun Ishida, Aya Harada, Takeaki Ishihara, Hikaru Kubota, Hiroki Kawaguchi, Ryohei Sasaki, Hiroshi Mayahara

**Affiliations:** ^1^ Division of Radiation Oncology Kobe Minimally Invasive Cancer Center Chuo‐ku Kobe Hyogo Japan; ^2^ Division of Radiation Oncology Kobe University Graduate School of Medicine Chuo‐ku Kobe Hyogo Japan; ^3^ Division of Radiation Therapy Kita‐Harima Medical Center Hyogo Japan; ^4^ Division of Radiological Technology Kobe Minimally Invasive Cancer Center Chuo‐ku Kobe Hyogo Japan; ^5^ Department of Radiology Kobe Minimally Invasive Cancer Center Chuo‐ku Kobe Hyogo Japan; ^6^ Division of Radiation Oncology Kobe University Hospital Chuo‐ku Kobe Hyogo Japan

**Keywords:** CyberKnife, fiducial marker, internal target volume, lung cancer, radiotherapy, stereotactic body radiotherapy

## Abstract

**Purpose:**

Real‐time tracking systems of moving respiratory targets such as CyberKnife, Radixact, or Vero4DRT are an advanced robotic radiotherapy device used to deliver stereotactic body radiotherapy (SBRT). The internal target volume (ITV) of lung tumors is assessed through a fiducial marker fusion using four‐dimensional computed tomography (CT). It is important to minimize the ITV to protect normal lung tissue from exposure to radiation and the associated side effects post SBRT. However, the ITV may alter if there is a change in the position of the fiducial marker with respect to the tumor. This study investigated the relationship between fiducial marker position and the ITV in order to prevent radiation exposure of normal lung tissue, and correct target coverage.

**Materials and methods:**

This study retrospectively reviewed 230 lung cancer patients who received a fiducial marker for SBRT between April 2015 and September 2021. The distance of the fiducial marker to the gross tumor volume (GTV) in the expiratory (*d*_ex_) and inspiratory (*d*_in_) CT, and the ratio of the ITV/V(GTV_ex_), were investigated.

**Results:**

Upon comparing each lobe, although there was no significant difference in the *d*_diff_ and the ITV/V(GTV_ex_) between all lobes for *d*_ex_ < 10 mm, there was significant difference in the *d*_diff_ and the ITV/V(GTV_ex_) between the lower and upper lobes for *d*_ex_ ≥ 10 mm (*p* < 0.05). Moreover, there was significant difference in the *d*_diff_ and the ITV/V(GTV_ex_) between *d*_ex_ ≥10 mm and *d*_ex_ < 10 mm in all lung regions (*p* < 0.05).

**Conclusion:**

The ITV that had no margin from GTVs increased when *d*_ex_ was ≥10 mm for all lung regions (*p* < 0.05). Furthermore, the increase in ITV tended to be greater in the lower lung lobe. These findings can help decrease the possibility of adverse events post SBRT, and correct target coverage.

## 1 INTRODUCTION

Stereotactic body radiotherapy (SBRT) facilitates the administration of large doses of radiotherapy per fraction and a small number of fractions to tumors with minimal exposure to the surrounding organs. It offers greater stability and accuracy, and is widely used for lung cancer treatment. Hypo‐fractionated high‐dose SBRT has emerged as the preferred treatment option for early‐stage non‐small cell lung cancer,^1^ as it is more efficient at avoiding radiation exposure to healthy lung tissue. However, movements associated with respiration, although minute, can interfere with accurate radiation delivery.^2^

The CyberKnife (Accuray, Sunnyvale, CA, USA) system is a frameless stereotactic radiosurgery system that tracks tumors in real‐time and allows patient movement during treatment; this system offers a fiducial marker‐based tracking with Synchrony™ or a fiducial‐free tracking with XSight Lung Tracking™ (XLT). The Synchrony technique utilizes internally placed fiducial markers around the target tumor for respiratory management.^3^ Real‐time tumor tracking during radiotherapy is used for treatment during respiratory movements.^4^ The implanted fiducial markers are safe and stable throughout the course of treatment for lung cancer.^2^, ^5^, ^6^, ^7^, ^8^, ^9^ However, adverse effects post SBRT often include radiation pneumonitis (RP), which can be fatal.^10^, ^11^, ^12^, ^13^, ^14^ Therefore, it is imperative to minimize unnecessary radiation exposure of normal lung tissue. In order to prevent adverse effects and control the tumor, it is important not to contour the target volume unnecessarily large, nor too small. To create an internal target volume (ITV), each phase of the four‐dimensional computed tomography (CT) image is fused with a fiducial marker. Then, the ITV is created by summation of all the gross tumor volumes (GTVs) obtained from each phase of the four‐dimensional CT image.^15^

To the best of the authors’ knowledge, there have been no studies conducted that investigate the relationship between the position of the fiducial marker and corresponding target volume. If the positional relationship between the fiducial marker and tumor changes, it is possible that the ITV will become large. In this study, the relationship between the fiducial‐marker position and ITV was investigated with the objective of minimizing the target volume to prevent unnecessary irradiation exposure of normal lung tissue in lung cancer patients.

## 2 METHODS

### 2.1 Patients

This retrospective study was conducted with the approval of the Institutional Review Board (reference 2021‐kenkyu07‐05). The fiducial‐marker position was analyzed in consecutive patients who underwent fiducial marker placement in preparation for SBRT for lung cancer based on the CyberKnife Synchrony Respiratory Tracking System (VSI, version 9.6.0) between April 2015 and September 2021.

A total of 230 patients (157 men and 73 women, including stage I non‐small cell lung cancer patients^16^), who underwent fiducial markers placement in the upper (*n* = 100), lower (*n* = 100), and middle (*n* = 50) lobes of the affected lung were investigated. These data were from a combination of both right and left lungs. These patients were selected in order from the latest date for the upper, lower, and middle lung lobe, respectively. Additional patient characteristics are presented in Table 1.

**TABLE 1:** Patient characteristics

Characteristics	All patients (*n* = 230)
Sex	
Male	157 (68%)
Female	73 (32%)
Age (years)	
Median (range)	76 (41–98)
Tumor site	
Upper lobe/lower lobe/middle lobe	100/100/50
V(GTV_ex_) volume (cm^3^)	
Median (range)	4.03 (0.15–39.76)
Mean (SD)	6.55 (7.51)
ITV volume (cm^3^)	
Median (range)	5.36 (0.31–52.73)
Mean (SD)	8.80 (9.58)
PTV margin (mm)	
Median (range)	4 (3–10)
Set‐up position	
supine/prone set‐up	232/18

Abbreviations: V(GTV_ex_), gross tumor volume in the expiratory phase; ITV, internal target volume; PTV, planning target volume; SD, standard deviation.

### 2.2 Fiducial placement

**FIGURE 1:**
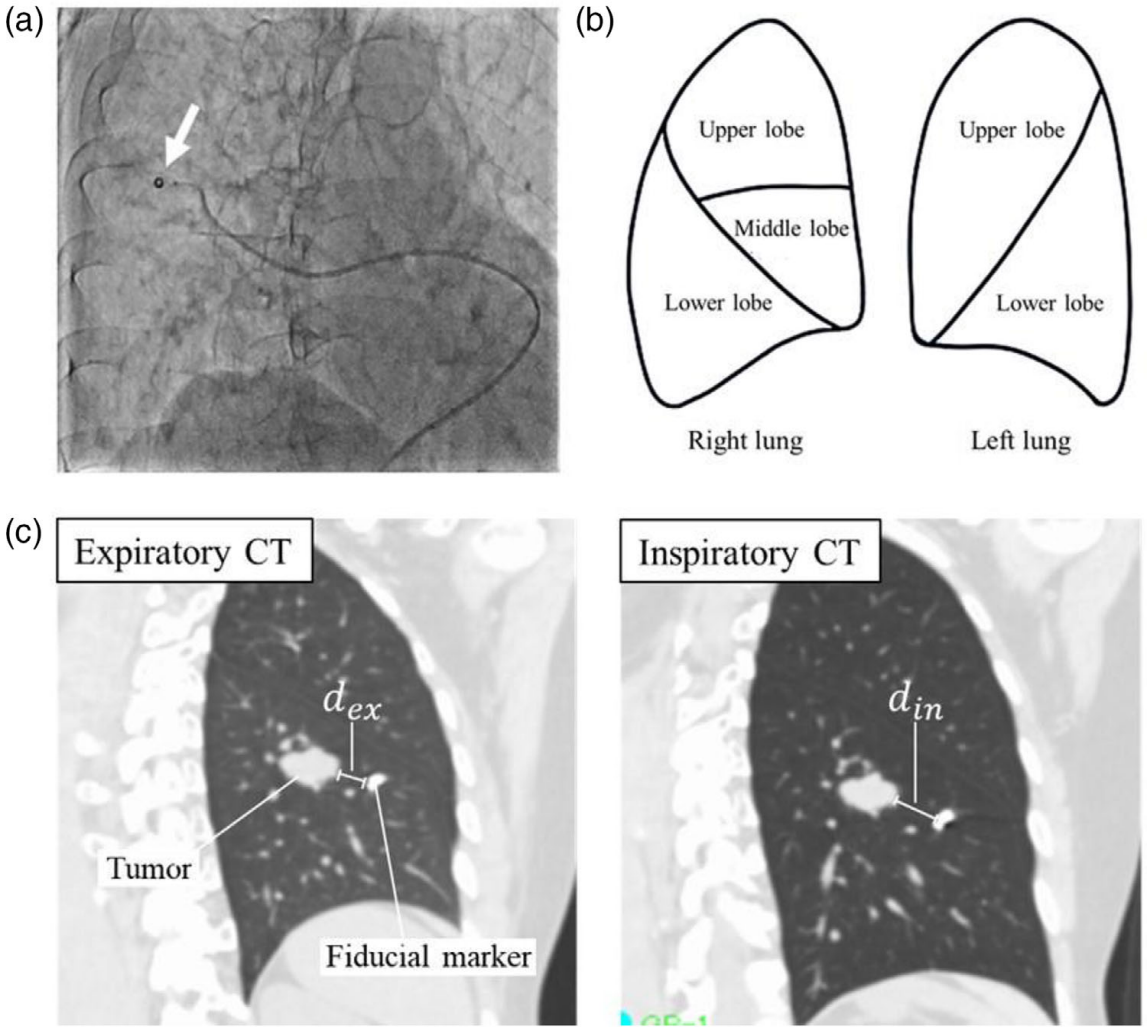
(a) Digital fluoroscopic image showing a microcatheter placed in a smaller pulmonary artery. The coil being pushed out of the catheter is indicated by a white arrow. (b) Schematic drawing of the upper, middle, and lower lobes in the right lung and the upper and lower lobes for the left lung that were used to divide the patients into different subgroups based on tumor location. (c) The distance of the fiducial marker to the gross tumor volume in the expiratory (*d*_ex_) and inspiratory (*d*_in_) computed tomography (CT) images are shown

Platinum microcoils with a 0.018‐inch diameter (Tornado Embolization Microcoil, Cook Medical, Indiana, USA) were implanted as fiducial markers. All fiducial markers were placed transarterially using an angio‐CT unit (INFX 8000C/JU and Aquilion LB, CANON Medical Systems, Tochigi, Japan). The size of the coils was chosen based on the target vessel size in our institution. First, a contrast‐enhanced CT was performed to confirm the positional relationship between the tumor and the pulmonary artery. The guide wire attached to the sheath was punctured from the femoral vein, and a 4.5‐Fr guiding sheath (Parent Plus 45, MEDIKIT CO., Ltd., Tokyo, Japan) was placed. Next, a catheter [5‐Fr 100 cm Headhunter type (HHA, MEDIKIT Co., Ltd., Tokyo, Japan) or 4‐Fr 110 cm Cobra type (C2, MEDIKIT Co., Ltd., Tokyo, Japan)] and the 0.035‐inch diameter guide wire (Radifocus, Terumo Co., Tokyo, Japan) were inserted into the sheath and advanced to the pulmonary artery via the right atrium and right ventricle. The pulmonary artery branch running near the tumor was selected as the final position of the catheter. After selecting the pulmonary artery branch, a CT scan was performed to confirm the position of the catheter. If the CT confirmed the absence of complications, a microcatheter (ASAHI Tellus, ASAHI INTECC Co., Ltd., Aichi, Japan) was inserted, and pulmonary arteriography was performed to confirm the vessel diameter. Then, a coil of the appropriate diameter was placed under fluoroscopy (Figure 1a). Finally, a CT scan was performed to confirm the positional relationship between the tumor and coil, and to detect possible complications such as alveolar hemorrhage. The maximum distance between the coil and tumor was no more than 50 mm, as recommended in a previous report.^17^

### 2.3 Radiation therapy

The Synchrony Respiratory Tracking System (Accuray, Sunnyvale, CA, USA) was used for real‐time tracking of the tumor. The CyberKnife planning platform (Precision version 2.0.1.1; CyberKnife (VSI) version 9.6.0) was used to create treatment plans. Patients were simulated in a supine or prone position and immobilized with a vacuum cushion (Vac‐Lok, CIVCO Medical Solutions, Coralville, IA, USA). Contrast‐enhanced CT images at the expiratory phase, inspiratory phase, and all phases of the four‐dimensional were performed to identify the ITV, and the expiratory scan was used to identify the GTV in the expiratory phase (V(GTV_ex_)). The clinical target volume (CTV) was equal to the GTV, as defined for each phase of respiration. ITV was obtained as a fiducial marker fusion of the CTV in both inspiratory phase CT images and all phases of four‐dimensional CT images with expiratory phase CT images, respectively, using Velocity AI 3.2.1 (Varian Medical Systems, Palo Alto, CA). These fusions did not allow any rotation or scaling. The planning target volume (PTV) was derived via the ITV using almost 4 mm margins in all directions in consideration of the correlation error, which was defined as the difference between the predicted and actual target positions in the Synchrony Respiratory Tracking System. The dose distribution was calculated based on expiratory CT images. During treatment, the respiratory fiducial marker motion was actively compensated for by the dynamic Synchrony Respiratory Tracking System.

### 2.4 Evaluation

The lungs were divided into three regions: the lower, upper, and middle lobes (Figure 1b). The minimum distance of the highest Hounsfield Unit point in the fiducial marker to the tumor border was measured if the fiducial marker was not placed inside the tumor. The difference in distance ($d_{\mathrm{diff}}$) was defined as follows:

$$d_{\mathrm{diff}}=\left|d_{\mathrm{ex}}-d_{\mathrm{in}}\right|,$$

where *d*_ex_ is the minimum 3D distance of the fiducial marker to the tumor border in the expiratory CT, *d*_in_ is the minimum 3D distance in the inspiratory CT, as shown in Figure 1c. The minimum 3D distance was measured in three dimensions using ShadeQuest/ViewR‐DG V1.27 (FUJIFILM Medical Solutions Corporation, Tokyo, Japan). The ITV/V(GTV_ex_) was defined to evaluate the extent of ITV expansion from the V(GTV_ex_).

### 2.5 Statistical analysis

Data are presented as the mean ± standard deviation (SD). All statistical analyses were performed with EZR software version 1.55 (Saitama Medical Center, Jichi Medical University, Saitama, Japan) on R commander version 2.7‐1.^18^ Differences between the groups were analyzed using the Kruskal–Wallis test with Bonferroni correlation or Mann–Whitney *U*‐test, and *p*‐value < 0.05 was considered statistically significant.

### 2.6 Financial support

This research received no specific grant from any funding agency in the public, commercial, or not‐for‐profit sectors.

## 3 RESULTS

### 3.1 Fiducial marker position and GTV_ex_ and ITV

**FIGURE 2:**
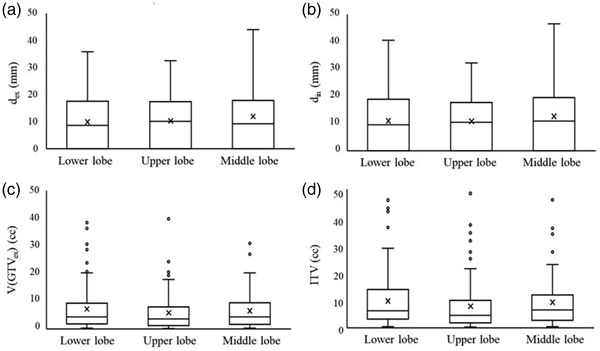
Quantitative comparison results for the distance of the fiducial marker to the gross tumor volume in the (a) expiratory (*d*_ex_) and (b) inspiratory (*d*_in_) phase computed tomography, (c) gross tumor volume in the expiratory phase (V(GTV_ex_)), and (d) internal target volume (ITV) in the lower, upper, and middle lung lobes

The box plots of expiratory (*d*_ex_) and inspiratory (*d*_in_) CT for each lung area (i.e., lower, upper, and middle lobes) are shown in Figure 2a,b, respectively. Similarly, the V(GTV_ex_) and ITV for each lung area (i.e., lower, upper, and middle lobes) are shown in Figure 2c,d, respectively. There was no significant difference among the areas for *d*_ex_, *d*_in_, the V(GTV_ex_), and ITV (Table 2).

**TABLE 2:** Parameters of *d*_ex_, *d*_in_, V(GTV_ex_) volume, and ITV

			*p*‐value		
Parameters	Region	Mean (SD)	Lower vs Upper	Lower vs Middle	Upper vs Middle
*d*_ex_ (mm)	Lower	9.91 (9.40)		> 0.05	
	Upper	10.14 (8.82)			
	Middle	11.99 (12.18)			
*d*_in_ (mm)	Lower	11.00 (10.32)		> 0.05	
	Upper	10.86 (9.10)			
	Middle	12.67 (12.63)			
V(GTV_ex_) (cc)	Lower	7.16 (8.33)		> 0.05	
	Upper	5.83 (6.68)			
	Middle	6.49 (7.32)			
ITV (cc)	Lower	9.65 (10.17)		> 0.05	
	Upper	7.77 (8.56)			
	Middle	9.17 (9.76)			

Abbreviations: *d*_ex_, expiratory computed tomography; *d*_in_, inspiratory computed tomography; V(GTV_ex_), gross tumor volume in the expiratory phase; ITV, internal target volume.

### 3.2 Evaluation of *d*_diff_ and ITV/GTV_ex_

**FIGURE 3:**
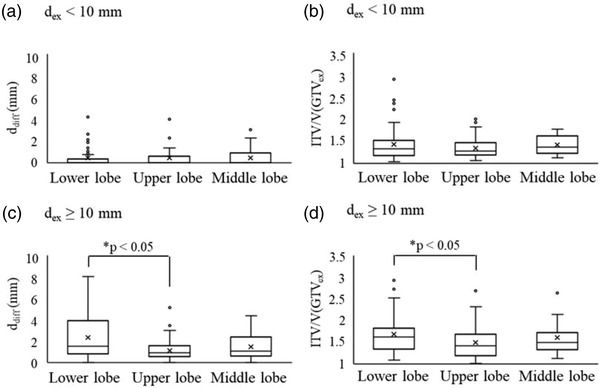
Quantitative comparison results for (a) the difference (*d*_diff_) between the distance of the fiducial marker to the gross tumor volume in the expiratory (*d*_ex_) and inspiratory (*d*_in_) phase computed tomography, and (b) the ratio of internal target volume (ITV) to gross tumor volume in the expiratory phase (V(GTV_ex_)) in each of the lower, upper, or middle lung lobes according to *d*_ex_ < 10 mm. Quantitative comparison results for (c) *d*_diff_ and (d) ITV/V(GTV_ex_) in each of the lower, upper, or middle lung lobes according to *d*_ex_ ≥ 10 mm

In case of *d*_ex_ < 10 mm, the box plots of *d*_diff_ and the ITV/V(GTV_ex_) for each lung area are shown in Figure 3a,. The mean *d*_diff_ were 0.43 mm (SD; 0.92 mm), 0.44 mm (SD; 0.90 mm) and 0.44 mm (SD; 0.82 mm), and the mean ITV/V(GTV_ex_) were 1.43 (SD; 0.38), 1.34 (SD; 0.24), and 1.42 (SD; 0.21) for the lower, upper, and middle lobes, respectively (Table 3). There was no significant difference in *d*_diff_ and the ITV/V(GTV_ex_) between the lower, upper, and middle lobes for *d*_ex_ < 10 mm. In case of *d*_ex_ ≥ 10 mm, the box plots of *d*_diff_ and the ITV/V(GTV_ex_) for each lung area are shown in Figure 3c,d. The mean *d*_diff_ were 2.36 mm (SD; 2.09 mm), 1.14 mm (SD; 0.95 mm), and 1.47 mm (SD; 1.17 mm) for the lower, upper, and middle lobes, respectively, and the mean ITV/V(GTV_ex_) were 1.69 (SD; 0.46), 1.49 (SD; 0.39), and 1.57 (SD; 0.30) for the lower, upper, and middle lobes, respectively (Table 3). The *d*_diff_ and the ITV/V(GTV_ex_) were larger for the lower lobe than for the upper lobe for *d*_ex_ ≥ 10 mm (*p* < 0.05; Table 3).

**TABLE 3:** Parameters of *d*_diff_ and ITV/V(GTV_ex_)

				*p*‐value					
							Lower	Upper	Middle
Parameters	*d*_ex_	Region	Mean (SD)	Lower vs Upper	Lower vs Middle	Upper vs Middle	< 10 mm vs ≥ 10 mm	< 10 mm vs ≥ 10 mm	< 10 mm vs ≥ 10 mm
*d*_diff_ (mm)	< 10 mm	Lower	0.43 (0.92)		> 0.05			< 0.05	
		Upper	0.44 (0.90)						
		Middle	0.44 (0.82)						
	≥ 10 mm	Lower	2.36 (2.09)	< 0.05		> 0.05			
		Upper	1.14 (0.95)						
		Middle	1.47 (1.17)						
ITV/V(GTV_ex_)	< 10 mm	Lower	1.43 (0.38)		> 0.05			< 0.05	
		Upper	1.34 (0.24)						
		Middle	1.42 (0.21)						
	≥ 10 mm	Lower	1.69 (0.46)	< 0.05		> 0.05			
		Upper	1.49 (0.39)						
		Middle	1.57 (0.30)						

Abbreviations: *d*_ex_, expiratory computed tomography; *d*_in_, inspiratory computed tomography; *d*_diff_, difference between *d*_ex_ and *d*_in_; V(GTV_ex_), gross tumor volume in the expiratory phase; ITV, internal target volume.

**FIGURE 4:**
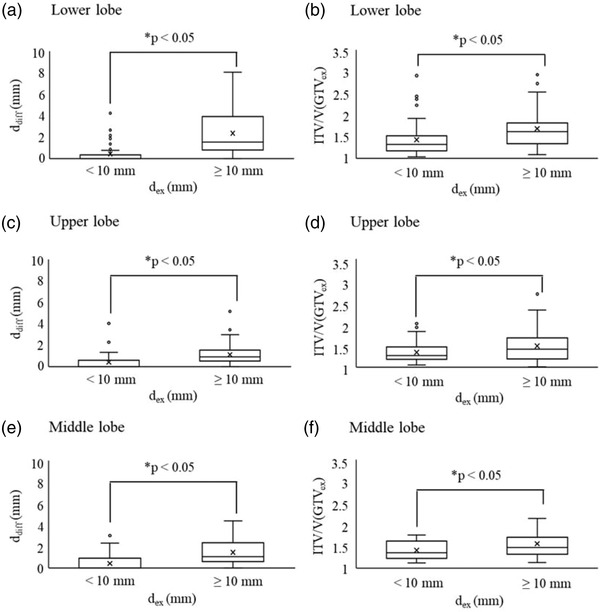
Quantitative comparison results for the difference (*d*_diff_) between the distance of the fiducial marker to the gross tumor volume in the expiratory (*d*_ex_) and inspiratory (*d*_in_) phase computed tomography and the ratio of internal target volume (ITV) to gross tumor volume in the expiratory phase (V(GTV_ex_)) in the lower [(a) and (b), respectively], upper [(c) and (d), respectively], and middle [(e) and (f), respectively] lung lobes grouped according to *d*_ex_ < 10 mm and ≥10 mm

In Figure 4, the box plots of *d*_diff_ and the ITV/V(GTV_ex_) for *d*_ex_ < 10 mm and ≥10 mm are shown for the lower, upper, and middle lobes, respectively. For all lung areas, the *d*_diff_ and the ITV/V(GTV_ex_) were larger for *d*_ex_ ≥ 10 mm than for *d*_ex_ < 10 mm (*p* < 0.05; Table 3).

**FIGURE 5:**
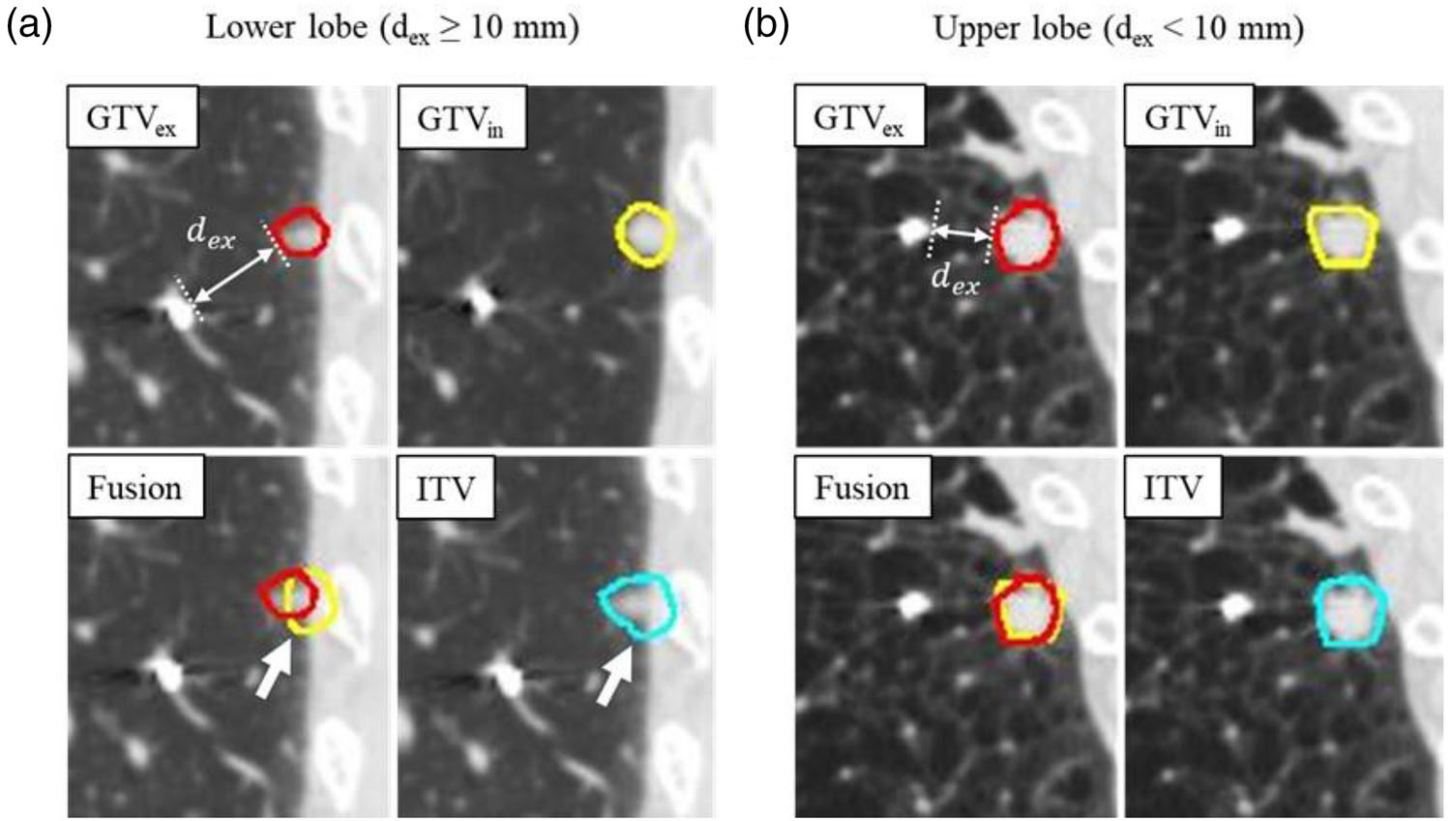
The delineations of the gross tumor volume in the expiratory phase (GTV_ex_), the GTV in the inspiratory phase (GTV_in_) and internal target volume (ITV) equal to the fusion of GTV_ex_ and GTV_in_ in (a) lower and (b) upper lung lobes are also shown for the same patient

Figure 5a,b shows the most variable case and the most stable case, respectively. In the most variable case (lower lobe and *d*_ex_ ≥ 10 mm), it was shown to increase the ITV compared to the V(GTV_ex_), as indicated by the white arrow. On the other hand, in the stable case (upper lobe and *d*_ex_ < 10 mm), it was shown almost the same ITV as the V(GTV_ex_).

## 4 DISCUSSION

The CyberKnife system is suitable for SBRT for lung tumors because of its highly concentrated dose administration and real‐time tumor tracking system, such as the Synchrony™ or XLT techniques. However, side effects like RP are frequently reported post SBRT, especially since the treatment is frequently performed on geriatric patients with co‐morbidities like chronic lung disease.^3^ To reduce the incidence of side effects after SBRT, the dose–volume histogram of the normal lung after SBRT^17^, ^19^, ^20^, ^21^ and the set‐up position of the patient during SBRT^22^ have been assessed.

Both the Synchrony™ and XLT techniques use a correlation between the respiratory phase and tumor position. XLT requires fulfillment of certain prerequisites, including a minimum tumor diameter of 15 mm, a tumor projection not aligned at 45° to the spine, and an adequate tumor density,^23^ Synchrony™ is flexible in that various tumor sizes, positions, and densities may be used as long as a fiducial marker can be placed. To minimize the target volume, we evaluated the relationship between the fiducial‐marker position and ITV in cases treated by the Synchrony technique, owing to its flexible nature. It is obvious that in the *d*_ex_ < 10 mm group, the ITV/V(GTV_ex_) was significantly smaller than that in the *d*_ex_ ≥ 10 mm group for each lung region. A general rule to implant fiducial markers with respect to the tumor was not established. Smith et al. noted that the correlation between the lung tumor and surrounding tissue typically deteriorates during lung movement in the superior/inferior direction. This is the largest component of the respiratory motion.^2^ Moreover, several researchers have reported that tumors in the lower lung lobes have the greatest motion.^24^, ^25^, ^26^ In particular, Knybel et al. reported that the mean motion amplitudes were significantly different in the lower and upper halves of the lungs (8.2 ± 4.2 mm and 3.6 ± 1.6 mm, respectively, *p* < 0.001).^27^ These reports support the results of the present study.

There was no significant difference in the *d*_ex_, *d*_in_, or the V(GTV_ex_) for each lung region, as shown in Figure 2 and Table 2, which indicate that these values were not biased in each lung region. However, the ITV compared with the V(GTV_ex_) may increase depending on the *d*_diff_ (Figures 3 and 4, and Table 3) because the shape changes between the expiratory and inspiratory phases in the lower lobe are greater than that in other lung regions, as mentioned by Nakao et al.^28^ The closer the fiducial marker is implanted to the tumor, the better it represents tumor motion, which decreases the influence of shape changes in the respiratory system. The threshold of *d*_ex_ was 10 mm for all regions in this study, and the ITV/V(GTV_ex_) increase was greater in the lower lung lobe.

In this study, fiducial placement was performed transarterially. In some cases, placing a fiducial marker near a peripheral lung tumor can be physically difficult. Hong et al. reported that percutaneous platinum endovascular embolization coils are much better than standard gold markers and are successfully tracked by the CyberKnife system.^29^ However, the percutaneous transthoracic method results in a high rate of pneumothorax.^30^, ^31^ Although transbronchial placement is another method for fiducial marker placement, this may be a difficult method to use for approaching peripherally located tumors, and might require moderate sedation, which can be a risk in patients with severe comorbidities.^32^ To avoid these events, transarterial placement of fiducial markers is a better alternative. Furthermore, transarterial fiducial marker implantation has several advantages. Although the evaluation of fiducial marker migration was not investigated in this study, Karaman et al. did not detect any migration of intravascular coils during follow‐up CT scans because these coils were fixed after placing them into a small blood vessel. Finally, they concluded that endovascular placement of coils as a fiducial marker is safe and feasible during CyberKnife therapy and might be an option for high‐risk patients who cannot undergo percutaneous transthoracic fiducial marker placement.^3^ Based on the literature, transarterial fiducial marker placement in this study was feasible for lung tumors treated with the CyberKnife system.

The PTV was created based on ITV. The margin of ITV‐to‐PTV was decided based on the value of the correlation error for each patient. This is because the targeting error of the CyberKnife radiation delivery system came from the correlation error between internal tumor locations versus external respiratory surrogate positions. Therefore, the correlation error affects the increase in PTV volume. The accuracy and ITV‐to‐PTV margin, based on the value of correlation error in the CyberKnife system, have been investigated by researchers.^28^, ^33^, ^34^, ^35^ Although the margin of ITV‐to‐PTV is always required, the PTV can be reduced by reducing the ITV, as mentioned in this study. Therefore, reducing the ITV leads to a reduction in adverse reactions after SBRT. In this study, the margin of GTVs‐to‐ITV was set to zero, while the ITV‐to‐PTV was set to almost 4 mm in all directions in the setting of four‐dimensional treatments using fiducials. If the four‐dimensional CT images cannot be used for creating an ITV or the setting of four‐dimensional treatments cannot be used for patients, the determination of the appropriate margin of GTV‐to‐PTV should be considered by both the correlation error and the amplitude of respiratory movement of lung tumor. In this study, the mean amplitude of respiratory movement of tumor was 6.5 mm for the upper lobe, 10.7 mm for the middle lobe, and 15.7 mm for the lower lobe. Sebastian et al. reported a set of data that is almost the same as ours.^36^ The user can refer to the above values to determine the appropriate margin of GTV‐to‐PTV in setting non‐four‐dimensional treatments using fiducials.

This study had some limitations. First, the patient's background and medical history, including the presence of co‐morbidities, were not considered. If the patient has some lung disease, it might affect the motion of the tumor, lung, or fiducial marker. Furthermore, the state of the tumor would also alter depending on its histology (e.g., squamous cell carcinoma, adenocarcinoma). Second, the set‐up position (supine/prone set‐up) was not considered. The set‐up position might affect the range of respiratory motion and the accuracy of real‐time tumor tracking by the CyberKnife system. In future studies, it is thus necessary to evaluate the patient background and set‐up position to understand the relationship between fiducial position and ITV more accurately.

## 5 CONCLUSIONS

We demonstrated that *d*_ex_ affects the ITV. Furthermore, the ITV increased significantly when *d*_ex_ was ≥10 mm for all lung regions, and the ITV increase was greater in the lower lung lobe. CyberKnife treatment is often performed in older patients or patients with co‐morbidities, such as chronic lung disease. In such patients, a reduction in the ITV is valuable to avoid adverse effects, such as RP, post SBRT. Meanwhile, the appropriate margin of GTVs‐to‐ITV or ITV‐to‐PTV should be set depending on whether four‐dimensional treatments using fiducials can be used or not. This is the first report to examine the relationship between the fiducial‐marker position and ITV. The findings of this report will be useful for physicians, radiation technologists, and medical physicists to decrease the possibility of adverse events after SBRT, and correct target coverage.

## CONFLICT OF INTEREST

The authors declare that there is no conflict of interest that could be perceived as prejudicing the impartiality of the research reported.

## AUTHOR CONTRIBUTION

Hiroaki Akasaka, Kazufusa Mizonobe, Yuya Oki, Kazuyuki Uehara, and Masao Nakayama were involved in study design and data interpretation. Shuhei Tamura, Yoshiki Munetomo, Haruna Kawaguchi, Jun Ishida, Aya Harada, Takeaki Ishihara, Hikaru Kubota, Hiroki Kawaguchi, and Ryohei Sasaki were involved in clinical treatment planning. Hiroaki Akasaka, Haruna Kawaguchi, Jun Ishida, and Hiroshi Mayahara were involved in data acquisition and analysis. All authors critically revised the report, commented on drafts of the manuscript, and approved the final report.
